# Functional Gene Diversity and Metabolic Potential of the Microbial Community in an Estuary-Shelf Environment

**DOI:** 10.3389/fmicb.2017.01153

**Published:** 2017-06-21

**Authors:** Yu Wang, Rui Zhang, Zhili He, Joy D. Van Nostrand, Qiang Zheng, Jizhong Zhou, Nianzhi Jiao

**Affiliations:** ^1^State Key Laboratory of Marine Environmental Science, Xiamen UniversityXiamen, China; ^2^Institute of Marine Microbes and Ecospheres, Xiamen UniversityXiamen, China; ^3^Institute for Environmental Genomics and Institute for Energy and the Environment and Department of Microbiology and Plant Biology, University of Oklahoma, NormanOK, United States; ^4^Earth Sciences Division, Lawrence Berkeley National Laboratory, BerkeleyCA, United States; ^5^School of Environment, Tsinghua UniversityBeijing, China

**Keywords:** GeoChip, East China Sea, functional gene, water mass, microbial community

## Abstract

Microbes play crucial roles in various biogeochemical processes in the ocean, including carbon (C), nitrogen (N), and phosphorus (P) cycling. Functional gene diversity and the structure of the microbial community determines its metabolic potential and therefore its ecological function in the marine ecosystem. However, little is known about the functional gene composition and metabolic potential of bacterioplankton in estuary areas. The East China Sea (ECS) is a dynamic marginal ecosystem in the western Pacific Ocean that is mainly affected by input from the Changjiang River and the Kuroshio Current. Here, using a high-throughput functional gene microarray (GeoChip), we analyzed the functional gene diversity, composition, structure, and metabolic potential of microbial assemblages in different ECS water masses. Four water masses determined by temperature and salinity relationship showed different patterns of functional gene diversity and composition. Generally, functional gene diversity [Shannon–Weaner’s *H* and reciprocal of Simpson’s 1/(1-*D*)] in the surface water masses was higher than that in the bottom water masses. The different presence and proportion of functional genes involved in C, N, and P cycling among the bacteria of the different water masses showed different metabolic preferences of the microbial populations in the ECS. Genes involved in starch metabolism (*amyA* and *nplT*) showed higher proportion in microbial communities of the surface water masses than of the bottom water masses. In contrast, a higher proportion of genes involved in chitin degradation was observed in microorganisms of the bottom water masses. Moreover, we found a higher proportion of nitrogen fixation (*nifH*), transformation of hydroxylamine to nitrite (*hao*) and ammonification (*gdh*) genes in the microbial communities of the bottom water masses compared with those of the surface water masses. The spatial variation of microbial functional genes was significantly correlated with salinity, temperature, and chlorophyll based on canonical correspondence analysis, suggesting a significant influence of hydrologic conditions on water microbial communities. Our data provide new insights into better understanding of the functional potential of microbial communities in the complex estuarine-coastal environmental gradient of the ECS.

## Introduction

Bacterioplankton are a crucial part of the marine food web and mediate major biogeochemical cycling in the ocean (Azam and Malfatti, 2007). They play an important role in organic matter decomposition (Herndl and Reinthaler, 2013), C and N fixation (Hügler and Sievert, 2010; Turk-Kubo et al., 2014), nitrification (Van Kessel et al., 2015), and denitrification (Ward et al., 2009). Microbial function in ecological and biogeochemical processes depends on the activity or behavior associated with their functional genes (Robinson et al., 2010). In *in situ* environments, the function of microbes is regulated by gene transcription as well as environmental variables. Meanwhile, the presence/absence and diversity of microbial functional genes suggest an associated functional potential in the ecosystem. Therefore, understanding the diversity of functional genes and uncovering the microbial functional potential are important for linking the microbial community to ecological and biogeochemical processes. In turn, the microbial community is inevitably influenced by the environment it inhabits. Some researchers have found associations of microbial communities with strong anthropogenic activity and natural gradients in complex coastal areas (Nogales et al., 2011; Ngugi et al., 2012). The close relationship between phylogenetic diversity of the microbial community and environmental factors such as temperature, salinity, and nutrients has been demonstrated in many marine ecosystems such as shelf and coastal areas (Baltar et al., 2015; Tinta et al., 2015; Lucas et al., 2016). Changes in environmental properties will influence microbial functional traits and consequently ecosystem function, which has been demonstrated in soil and marine environments (Thompson et al., 2016; Zhang et al., 2016). However, little is known about the functional gene composition and metabolic potential of bacterioplankton in estuaries areas, environmentally complex but ecologically important ecosystems.

The ECS is a marginal sea in the northwest Pacific Ocean. The KC flows along its eastern edge and brings its high-temperature water from the western equatorial Pacific. The TWC flows from the south along the eastern side of the sea, while the Yellow Sea Coastal Water flows from the north along the northwest coast of the sea (Tsai et al., 2010). The ECS is greatly influenced by the Changjiang River (YZR), which empties into the ECS from the west with decreasing salinity and conveys high levels of nutrients (Zhang et al., 2007), and is also influenced by oligotrophic, high salinity oceanic waters from the east (Jiao et al., 2005). Depending on the hydrographic stages of the YZR, nutrient and chlorophyll *a* concentrations generally decrease seaward from estuarine and coastal areas (Zhang et al., 2007). The hydrographic complexity of the ECS and the concomitant heterogeneous temperature, nutrient, and salt concentrations result in dynamic environmental conditions, which have profound impacts on the microbial communities in this environment (Schultz et al., 2003; Kirchman et al., 2005; Fuhrman et al., 2006).

Studies have indicated that the changes in temperature, salinity, and nutrients from the YZR discharge and KC result in different distribution patterns of *Prochlorococcus, Synechococcus*, and heterotrophic bacteria in the ECS (Jiao et al., 2005), while freshwater influx and oligotrophic conditions impact the phylogenetic diversity and structure of bacterial and archaeal communities (Zeng et al., 2006; Hu et al., 2011, 2013). However, little is known about how the changes in environmental parameters affect the functional potential of the ECS microbial communities.

In this study, we analyzed microbial communities from different depths of the ECS using a functional gene microarray (GeoChip) to characterize differences in the functional potential based on location and depth. Specifically, we intended to address the following questions: (i) are there differences in the functional potential of the microbial community among the different water masses? (ii) Which functional processes change among water masses? And (iii) are there correlations between the functional potential of the microbial community and the properties of the water masses? Significant differences were observed in the overall functional diversity and in specific functional categories, such as carbon fixation, carbon degradation, and other key metabolic pathways, which were likely driven by temperature, salinity, and Chl *a* differences. This study provides new insights into our understanding of the functional diversity, structure, and metabolic potential of microbial communities in the ECS ecosystems.

## Materials and Methods

### Sampling

**FIGURE 1:**
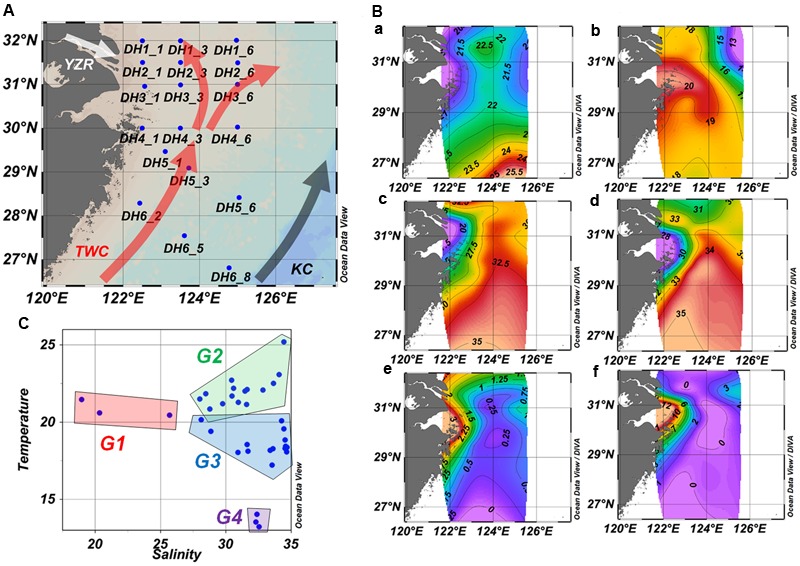
Sampling station **(A)**, environmental parameter distribution **(B)**, and temperature–salinity relationship **(C)** in the ECS. **(B)** Temperature (°C, a,b), salinity (c,d), and Chl *a* concentration (μg/L, e,f) of surface water samples (a, c, e) and bottom water samples (b, d, f). TWC, Taiwan warm current; KC, Kuroshio Current; YZR, Changjiang River.

Sampling and environmental measurements were conducted on board the research vessel *Kexue #3* in June 2010. Salinity, temperature, and conductivity were measured by a CTD (conductivity-temperature-depth) profiler (SeaBird Electronics, Inc., Bellevue, WA, United States) mounted on a rosette sampler equipped with 24 12-L Niskin bottles and a fluorometer for measuring chlorophyll *a* concentration. The ambient seawater samples (2 L) from surface layers (2 m) and layers near the seafloor (bottom, from 18 to 115 m) were collected at 21 sites along the western ECS coastal area to the edge of the continental shelf (**Figure 1A** and Supplementary Table S1). Water samples were filtered through two pre-filters, a 20 μm filter followed by a 3 μm filter, to remove large particles and then filtered through a 0.22 μm filter (GTTP, Millipore, Billerica, MA, United States) to collect microorganisms. The filters were stored at -80°C until DNA extraction.

### DNA Extraction and GeoChip Hybridization

The 0.22 μm filters were thawed and then cut into small fragments with a sterile scalpel. The fragments were then transferred to a 2 mL sterile tube, and 490 μL sucrose lysis buffer (20 mM EDTA, 400 mM NaCl, 0.75 M sucrose, 50 mM Tris-HCl, pH 9.0) (Venter et al., 2004) and 500 μL TE buffer (pH 8, containing 150 μg/mL lysozyme) were added. The tubes were incubated at 37°C for 1 h, and then 10 μL 10% SDS (final concentration 1%) was added. The tubes were then frozen in liquid nitrogen for 3–4 min, transferred to 65°C until thawed completely, and then frozen again in liquid nitrogen. This freeze-thaw cycle was performed three times. The resulting lysate was treated with 5 μL 20 mg/mL Proteinase K (final concentration 100 μg/mL) for 1 h at 55°C followed by two aqueous phenol extractions and one extraction with phenol/chloroform. The supernatant was then precipitated with 0.6 volumes of isopropanol and 0.1 volumes of 3 M sodium acetate, and then the DNA pellet was washed with 70% ethanol. Then DNA was re-suspended in 50 μL sterilized water. The final DNA concentrations were assessed using PicoGreen (Ahn et al., 1996) with a FLUOstar Optima (BMG Labtech, Jena, Germany).

To prepare the sample for hybridization, the DNA (∼10 ng) was amplified using whole community genomic amplification (Wu et al., 2006). Then, 3.0 μg of amplified DNA was labeled with Cy-3 and hybridized with the GeoChip 4.2 on a Hybridization Station (MAUI, BioMicro Systems, Salt Lake City, UT, United States) at 42°C for 16 h (Tu et al., 2014). The hybridized arrays were scanned using an MS 200 Microarray Scanner (NimbleGen, Madison, WI, United States).

### GeoChip Data Pre-processing

GeoChip 4 contains 82,000 oligonucleotide probes targeting 141,995 genes in 410 categories covering major microbial functional categories and biogeochemical functions, such as carbon, nitrogen, sulfur, and phosphorus cycling, metal resistance, and organic remediation (Tu et al., 2014). All GeoChip hybridization data were uploaded to an in-house data analysis pipeline^1^. The signal intensity of each probe was normalized for each sample as described previously (He et al., 2014), and spots with signal-to-noise ratio of less than 1.5 were removed. The signal intensity of each spot was then divided by the mean intensity of positive spots on the microarray to normalize the intensity of each spot. Probes detected in less than two samples were removed. The GeoChip data was deposited at the IEG website^2^ and PANGAEA (doi: 10.1594/PANGAEA.875049).

### Statistical Analysis

Four different diversity indices [Shannon–Weiner’s *H*, reciprocal of Simpson’s 1/(1-*D*), Simpson evenness *Si*, and Pielou’s evenness *J*] were used to calculate the functional gene diversity based on the all detected probes. The proportion of functional category/subcategory/gene detected was calculated as the sum of detected probe numbers in each functional category/subcategory/gene in one sample divided to the total detected probe number in the same sample. Statistical differences in the mean proportion of gene in each functional category and subcategory among different water masses were analyzed by a one-way ANOVA and Tukey’s test as a *post hoc* test. A significance level of *P* < 0.05 was adopted for all comparisons with a 95% confidence interval (He and Wang, 2011). The sum of the normalized intensity values (total abundance) for each gene category or family was used for ANOVA. PERMANOVA was performed in this study, based on the Bray–Curtis distance of the proportion of all probes detected. Multivariate analyses of GeoChip data, including detrended correspondence analysis for comparing the different functional gene communities (Zhou et al., 2008) and canonical correspondence analysis and the Mantel test for linking microbial communities to environmental variables, were employed. In addition, the Pearson correlations were calculated between diversity indices with environmental variables. Water mass environmental variables were analyzed by principal component analysis. The comparison of environmental variables between in each water mass were conducted by Mann–Whitney Rank Sum Test since the homoscedasticity of the data was not met. All analyses were performed using the *vegan* package in R 3.0.2 (R Core Team, 2013).

## Results

### Characterization of Sampling Sites

The horizontal and vertical profiles of temperature, salinity, and Chl *a* indicated a clear influence from the YZR input and KC on the ECS (**Figure 1B**). The results showed low salinity and temperature near the mouth of the YZR, and high temperature at the south of the ECS. The mean temperature (mean ± standard deviation, 21.83 ± 0.23°C) of surface samples (*n* = 21) was significantly higher than that (18.62 ± 1.26°C) of the bottom samples (*n* = 15, Mann–Whitney Rank Sum Test, *P* = 0.001), while salinity showed the reverse pattern (surface vs. bottom sample: 29.52 ± 0.86 vs. 34.17 ± 1.38, Mann–Whitney Rank Sum Test, *P* = 0.001). These results demonstrated that the YZR brought low-salinity but high-nutrient water into the ECS, while the KC and TWC transported warm and low-nutrient equatorial water from the equatorial western Pacific, consistent with the findings of a previous study (Tian et al., 1993). The Chl *a* concentration ranged from 0.5 to 11.5 μg/L over the study area (Supplementary Table S1). The sites near the mouth of the YZR showed relatively high Chl *a* concentration (**Figure 1B**). These results revealed the complex hydrography and environmental conditions in the ECS.

The temperature–salinity relationship was used to identify the different water masses (Emery and Meincke, 1986). Based on the temperature–salinity relationship, we identified four different groups of samples corresponding to different water masses (**Figure 1C**): Group 1 (G1) contained surface samples with low temperature (from 20.46 to 21.47°C) and salinity (from 18.96 to 25.69) collected from near the YZR mouth; Group 2 (G2) included the other surface samples with high temperature (from 20.81 to 25.20°C) and moderate salinity (from 28.01 to 34.45), which were influenced by the TWC and KC; Group 3 (G3) contained almost all of the bottom samples, characterized by moderate temperature (from 18.03 to 19.57°C) and high salinity (from 28.85 to 34.68); Group 4 (G4) was composed of three bottom samples with low temperature (from 13.23 to 14.03°C) and high salinity (from 32.30 to 32.54), which were collected from locations at the northwest of the ECS. Principal component analysis based upon the longitude, latitude, depth, temperature, salinity, and Chl *a* showed the distinct differences among the four water-mass groups (Supplementary Figure S1).

### Overall Functional Gene Diversity and Structure of Microbial Communities

The number of phylotypes that were detected for each microbial functional gene by the GeoChip ranged from 4,000 to 14,000 per sample (Supplementary Table S1). A significant difference in functional gene diversity indices [*H* and 1/(1-*D*)] was detected among the four groups of microbial communities based on a Kruskal–Wallis one-way ANOVA (*P* < 0.001), and the Dunn’s test showed a significant difference between G2 and G3 [*H* and 1/(1-D), *P* < 0.001 and 0.001, respectively] (**Table 1**). Moreover, the average *H* and 1/(1-*D*) of surface waters were higher than those of the bottom waters (Mann–Whitney test, *P* < 0.001 and 0.001, respectively). These results indicated a higher diversity of functional genes in the microbial community of the surface seawater of the ECS. PERMANOVA also showed a significant difference in functional gene structure between surface and bottom samples (**Table 2**). In addition, detrended correspondence analysis results revealed a clear difference in functional gene structure among the microbial communities of the different water masses (Supplementary Figure S2).

**Table 1:** Diversity indices based on all probes detected in the ECS.

	*H*	*SD*	1/(1-*D*)	*SD*	*J*	*SD*	*Si*	*SD*	*n*
G1	9.1927	0.1052	9840.504	1033.2	0.9996	1.39E-05	0.9922	2.82E-04	3
G2	9.2682	0.2332	10833.08	2293.742	0.9997	5.93E-05	0.9940	1.19E-03	18
G3	9.012	0.4292	8934.415	3643.898	0.9997	7.24E-05	0.9941	1.46E-03	12
G4	8.969	0.0752	7855.736	583.4106	0.9997	5.17E-05	0.9944	9.63E-04	3
Surface	9.261	0.2252	10738.55	2224.127	0.9997	6.35E-05	0.9939	1.26E-03	21
Bottom	9.0034	0.3857	8718.679	3297.975	0.9997	6.91E-05	0.9941	1.38E-03	15

*H denotes Shannon–Weiner index, 1/(1-*D*) denotes reciprocal of Simpson’s index, *J* denotes Pielou’s evenness, and *Si* denotes Simpson evenness. *SD* denotes standard deviation.*

**Table 2:** Permutational multivariate analysis of variance based on Bray–Curtis dissimilarity among sample groups in the ECS.

	G1	G2	G3	G4
G1		0.001	0.001	0.001
G2	6.116		0.001	0.002
G3	6.561	21.726		0.001
G4	9.590	11.638	4.845	

*Upper triangle is the significance *P*-value; lower triangle is the *R* square of PERMANOVA.*

**FIGURE 2:**
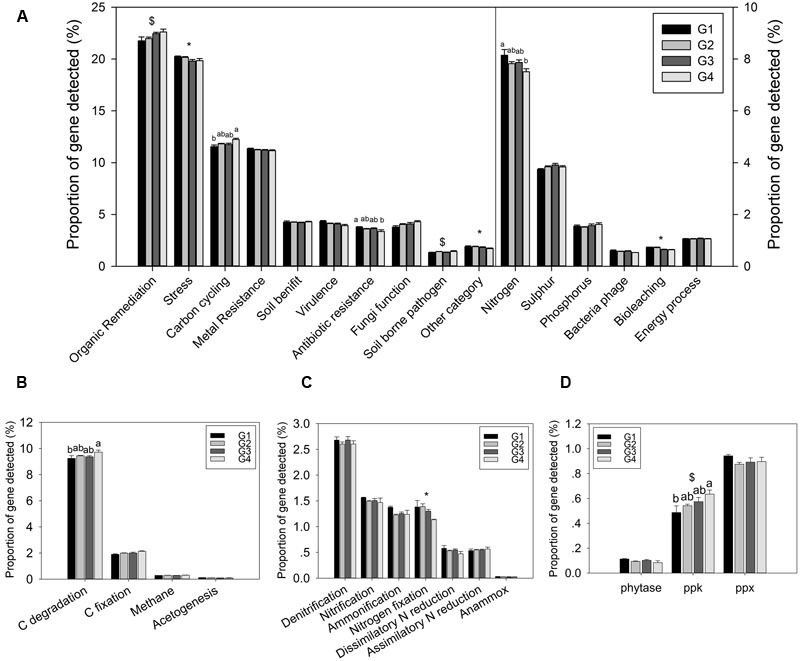
Proportions of genes in different functional categories. **(A)** All functional gene categories, **(B)** C-cycling subcategories, **(C)** N-cycling subcategories, and **(D)** P-cycling subcategories. All data are shown as mean ± standard deviation of the proportions of each gene category, or each subcategory, of microarray probes detected in each group of samples in the ECS. Significant differences among/between those groups, and between surface and bottom water samples indicated by ANOVA with Tukey’s *post hoc* test are indicated by letters a and b, and by ^∗^ and $, separately, above the bars. The different letters indicate *P* < 0.05. ^∗^ Indicates higher mean proportion in surface samples, while $ indicates higher mean proportion in bottom samples with *P* < 0.05.

The proportion of genes involved in major biogeochemical cycling was determined in this study. Three functional categories of genes showed a significant difference in proportion among the four groups: C cycling, N cycling, and antibiotic resistance (**Figure 2A**). However, most of functional categories and subcategories showed not significant differences among the four groups. These results suggested a certain functional heterogeneity of the microbial communities across the ECS shelf.

### Functional Genes Involved in Biogeochemical Cycling

**FIGURE 3:**
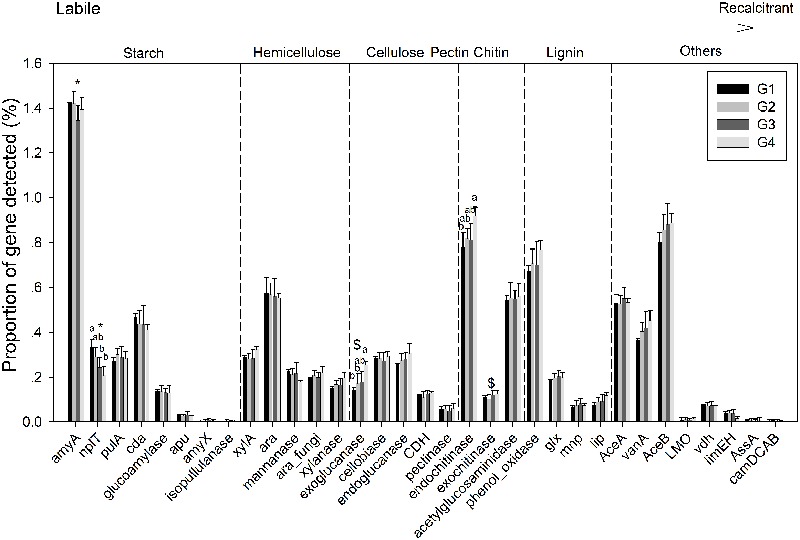
Proportions of each gene within the C-degradation subcategory. Significant differences among/between the groups, and between surface and bottom samples, indicated by ANOVA with Tukey’s *post hoc* test are indicated by letters a and b, and by ^∗^ and $, separately, above the bars. The different letters indicate *P* < 0.05. ^∗^Indicates higher mean proportion in surface samples, while $ indicates higher mean proportion in bottom samples with *P* < 0.05.

**FIGURE 4:**
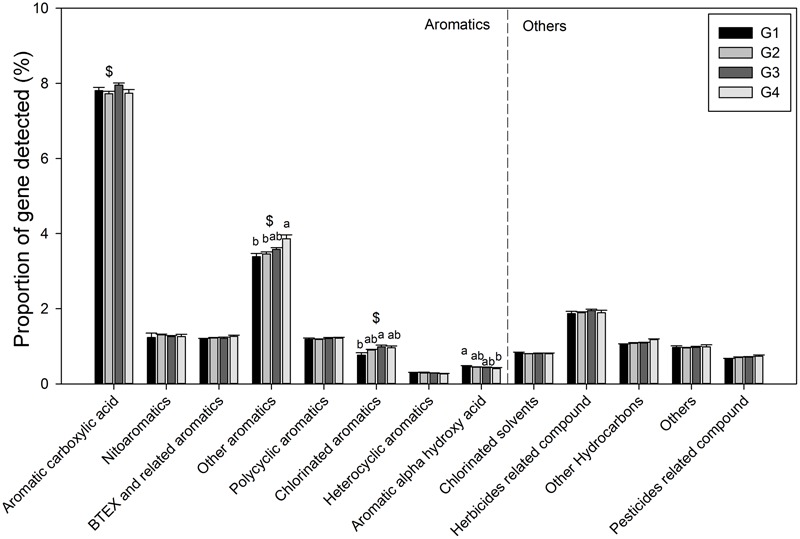
Proportions of each subcategory within organic remediation. All data are shown as mean ± standard deviation. Significant differences between the groups, and between surface and bottom samples, indicated by ANOVA with Tukey’s *post hoc* test are indicated by letters a and b, and by ^∗^ and $, separately, above the bars. The different letters indicate *P* < 0.05. ^∗^Indicates higher mean proportion in surface samples, while $ indicates higher mean proportion in bottom samples with *P* < 0.05.

Gene categories involved in major biogeochemical processes were analyzed to understand the functional distribution of microbial communities in the ECS (**Figure 2**). Key genes involved in C cycling were detected in all four groups, including C degradation, C fixation, and methane metabolism (**Figure 2B**). Among these, the proportion of C-degradation genes was significantly higher in G4 compared with G1. The average proportion of genes associated with starch metabolism, such as cytoplasmic alpha-amylase (*amyA*) and neopullulanase (*nplT*), was higher in the microbial communities at the surface than those at bottom of the ECS (**Figure 3**). In contrast, the genes encoding exoglucanase, as well as endochitinase and exochitinase, which are associated with cellulose and chitin degradation, showed higher proportion in the microbial communities from the bottom water compared with those from the surface water. Moreover, the genes involved in organic remediation were detected (**Figure 4**). The results showed that the proportion of genes involved in aromatic carboxylic acid, chlorinated aromatic, and other aromatics were higher in the bottom water. Meanwhile, the proportion of genes associated with chlorinated aromatics and other aromatics metabolism in G4 were significant higher than those in the G1 or G2.

Nitrogen is one of the key variables controlling ecosystem properties and functions, and marine microorganisms mediate all major biogeochemical transformations in the nitrogen cycling processes (Zehr and Kudela, 2011). Key genes for ammonification, anammox, assimilatory N reduction, denitrification, dissimilatory N reduction, nitrification, and nitrogen fixation were detected in the ECS (**Figure 2C**). The proportion of genes involved in N fixation (*nifH*) showed a significant difference between the microbial communities from the surface and bottom samples, suggesting a higher potential for N fixation in the surface water. In addition, two genes, *gdh* (*t* = 7.18, *P* = 0.011) involved in ammonification and *hao* (*t* = 6.89, *P* = 0.012) involved in nitrification, also showed a higher proportion in the microbial communities from the surface water. Meanwhile, a key gene involved in denitrification, *nirS*, showed a higher proportion (*t* = 4.34, *P* = 0.044) in the microbial communities from the bottom water; however, no significant differences were found for other genes involved in N cycling such as *ureC, hzo*, and *nasA* (Supplementary Table S2).

While three key genes (phytase gene, *ppk, ppx*) for P metabolism were detected, one-way ANOVA showed that only the proportion of the *ppk* gene, encoding polyphosphate kinase involved in oxidative phosphorylation, was significantly higher in G4 than in G1 microbial communities (**Figure 2D**). Additionally, a higher proportion of this gene was detected in the microbial communities from the bottom water samples (G3 and G4) than in those from the surface water samples (G1 and G2). A higher proportion of genes involved in phosphate limitation was observed in microbial communities from the surface water samples (Supplementary Figure S3), suggesting greater P limitation in the surface water (Karl, 2014).

### Relationship between Microbial Functional Diversity and Water Masses

**FIGURE 5:**
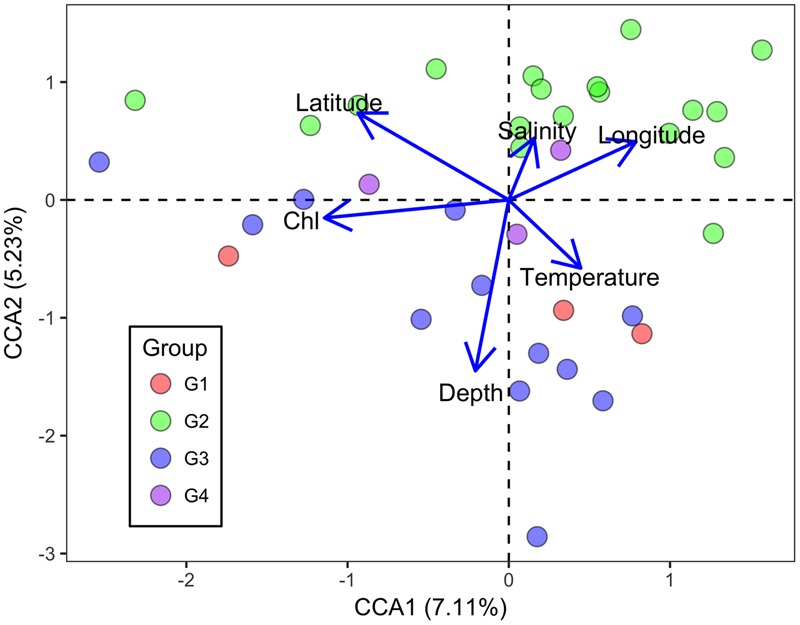
Canonical correspondence analysis of all genes detected with environmental parameters in the ECS. Blue and purple symbols denote bottom samples, while orange and green symbols denote surface samples. Sal denotes salinity, Temp denotes temperature, and Chl denotes chlorophyll *a* concentration.

Canonical correspondence analysis was performed to identify possible linkages between the microbial functional gene structure and environmental parameters (**Figure 5**). The first axis was positively correlated with temperature and negatively correlated with Chl *a*. The second axis was negatively correlated with Chl *a* and temperature, and positively correlated with salinity. Moreover, Chl *a* had an overall significantly negative correlation with the overall *H* (Pearson correlation coefficient = -0.451, *P* = 0.005) and 1/(1-D) (Pearson correlation coefficient = -0.464, *P* = 0.004). In the surface water, Chl *a* correlated negatively with *H* (Pearson correlation coefficient = -0.647, *P* = 0.001) and 1/(1-*D*) (Pearson correlation coefficient = -0.614, *P* = 0.002), while it correlated positively with evenness indices (*J* and *Si*) in the bottom samples (Pearson correlation coefficient = 0.487, *P* = 0.050; coefficient = 0.511, *P* = 0.043). Overall, a relationship between microbial communities and Chl *a* was observed, suggesting an impact of autotrophs on the functional gene structure of the microbial communities, although the extent of the influence varied between surface and bottom seawaters.

## Discussion

The GeoChip results indicated the complexity of the functional gene structure of the microbial community in the ECS. A previous study observed the effect of the currents, including TWC, KC, and YZR input, on the distribution of picoplankton in the ECS (Jiao et al., 2005). However, the current study did not observe a distinct influence from these currents on the microbial community functional potential in the surface water. Temperature is known to be an important factor affecting chemical and biochemical processes, and subsequently the diversity of microbial communities in marine ecosystems (Pomeroy and Wiebe, 2001). Higher temperature always facilitates a richer microbial community structure on a geographical scale (Pommier et al., 2007; Fuhrman et al., 2008). Previous studies have also demonstrated that salinity is a crucial determinant for microbial communities (Lozupone and Knight, 2007), and that the microbial phylogenetic distribution significantly varies along a salinity gradient in coastal waters (Campbell and Kirchman, 2013). The low temperature and salinity association of G1 resulted from the YZR input, while G2 with high temperature and salinity was strongly influenced by the TWC, and the Kuroshio-branched water intruded into the ECS shelf via the bottom layer (Lian et al., 2016). The temperature and salinity were spatially similar near the seafloor (Wang and Oey, 2016), suggesting a more stable environment in the deep seawater compared with the surface water. The positive correlation between salinity and GeoChip data suggested that, similar to the phylogenetic diversity, the functional gene diversity of the microbial communities increased as the salinity increased from coastal to open ocean. In addition, the abundance of heterotrophic bacteria in the surface and deep seawater did not vary much (Jiao et al., 2005). Together, temperature and salinity, which are determined by the water mass, showed a considerable influence on the functional gene structure of water microbial communities in the ECS.

Additionally, our data indicates that the functional potential of water microbial communities has a close relationship with primary production. Two high-Chl *a* areas were observed in our study, one was near the mouth of the YZR, another was offshore in the northeast, which was mostly consistent with previous observations (Guo et al., 2014). It is likely that high primary production reduced the functional gene diversity of microbial communities in the surface seawater. The decreased diversity of microbial communities caused by increased dissolved organic matter with the bloom has been demonstrated in several studies (Larsen et al., 2004; Landa et al., 2016). Here, we found that the functional gene evenness based on all detected genes in the bottom samples showed a significantly positive correlation with Chl *a* in the bottom seawater. Thus, the high primary production indicated by high Chl *a* might benefit the functions of microbial communities with low abundance in the bottom seawater. In contrast, there was no significant correlation between Chl *a* and functional gene evenness in the surface seawater samples (Pearson correlation *P-*value of *J* and *Si*, 0.098 and 0.085 separately). This different response to Chl *a* between surface and bottom microbial communities could be due to the heterogeneity of the microenvironment and composition of microbial communities in the bottom water compared with the surface water. The distinct structure of microbial communities between surface and bottom seawater have been demonstrated in various coastal areas, such as Pearl River estuarine (Liu et al., 2015), South China Sea (Zhang et al., 2014b), and Oregon and Washington coastal (Fortunato et al., 2012). Here, the greater degradation potential of the relatively recalcitrant C and aromatics was observed in bottom samples, especially in G4, which had high Chl *a* (**Figure 3**). Furthermore, higher proportion of bacterial groups that utilized the high-molecular-mass substrates such as *Gammaproteobacteria, Cytophaga*, and *Flavobacteria* (Cottrell and Kirchman, 2000; McCarren et al., 2010; Sosa et al., 2015) were often observed in the bottom seawater (Zhang et al., 2014b; Liu et al., 2015). Therefore, the increased functional gene evenness of microbial communities might be associated with the microbes able to utilize the high-molecular-mass substrates, which may be stimulated by primary production.

An eddy usually generated by the detachment from the YZR diluted water southwest of Jeju Island (∼125°E, 32°N), and the stratification was completely destroyed in the entire water column (Moon et al., 2010). In the current study, low temperature and salinity were observed at three sites (DH1-6, DH2-6, and DH3-6) at the northeast of the ECS near Jeju Island (**Figure 1B**), which was consistent with the detachment demonstrated previously (Moon et al., 2010). The resultant upwelling drew nutrients from the deep seawater and sediment, consequently increasing the amount of Chl *a*. Consistently, a high Chl *a* concentration was observed in this area in the present study (**Figure 1B**). Comparing the surface samples with bottom samples at these three sites, DH3-6 with low salinity showed a similar functional gene structure of water microbial communities at both depths (Supplementary Figure S4), which suggested a vertical mixing in this area. Overall, Chl *a*, the distribution of which was associated with the YZR discharge that caused the eddy, had a crucial impact on the functional gene diversity and evenness of the microbial community.

Previous studies of the ECS found that the phylogenetic diversity in the bottom bacterial community was higher than that in the surface bacterial community, especially along the outer edge of the shelf (Dong et al., 2014), although there was no significant difference in bacterial abundance between the surface and bottom seawater (Jiao et al., 2005). In contrast, we found the higher functional gene diversity in the surface samples than that in the bottom samples (**Table 2**). These results, however, suggested that a higher phylogenetic diversity did not accompany higher functional gene diversity. The inconsistency between the functional and phylogenetic diversities might suggest the functional redundancy of the microbial community within the ecosystem. According to the functional redundancy theory, different species in similar niches might play similar functional roles in biogeochemical cycling, and overlapping niches may increase the functional redundancy of the ecosystem. Indeed, another study showed that the phylogenetic diversity only partially correlated with functional diversity (Flynn et al., 2011). Furthermore, stable ecosystems generally have lower diversity (Wohl et al., 2004), which may in turn indicate a low functional diversity. The complex hydrography in the ECS area, where the YZR, KC, and TWC provide an influx of various organic matter, could disturb the stability of the ecosystem (Tsai et al., 2010; Yu et al., 2012; Dong et al., 2014). As such, surface bacterioplankton communities might have developed diverse functions to fully utilize the available heterogeneous organic matter and nutrients. Compared with the complex surface seawater environment, the bottom environment is more stable (Jiao et al., 2002, 2005). Despite the relatively higher concentration of nutrients in the bottom seawater (Yu et al., 2012), the relatively low concentration of labile substrate for bacterioplankton might reduce the functional gene diversity. In addition, the different copy numbers of some functional genes in bacterial genomes, such as the *amo* gene in different ammonia-oxidizing bacteria (Hommes et al., 2001), might also contribute to this inconsistency.

In the ECS marginal shelf, a large amount of labile and recalcitrant organic compounds is discharged by YZR runoff all year round, especially during the summer (Zhang et al., 2003). In addition, phytodetritus and zooplankton fecal pellets provide another source of organic matter in this system (Bertilsson and Jones, 2003; Polz et al., 2006). Hence, the vertical distribution of organic matter may influence the metabolic preference of microbes in the ESC. The genes involved in C degradation showed different distributions in microbial communities from surface water and bottom water (**Figures 2B, 3**). Since cellulose and chitin are more recalcitrant than starch (You et al., 2012; Hamre et al., 2014), these results indicate that surface bacterioplankton have a higher metabolic potential for utilizing relatively labile organic matter, while bottom bacterioplankton have a higher potential for degradation of relatively recalcitrant organic matter. The degradation of labile organic matter preferentially occurs in the surface seawater where it is generated or transferred, while the recalcitrant organic matter settles to the bottom and is buried in the sediment (Hulthe et al., 1998). Besides, the mixing along the ECS coast would introduce more of the aged sedimentary organic matter into the water column (Hulthe et al., 1998), where the bottom microbial communities are able to utilize it. Additionally, relatively aerobic conditions near the bottom would further enhance the degradation of the recalcitrant organic matter (Hulthe et al., 1998). A higher proportion of genes involved in organic remediation was also observed in the microbial communities of the bottom seawater (**Figure 2A**). Genes involved in aromatic metabolism (such as genes for metabolism of aromatic carboxylic acid and chlorinated aromatics) were the major contributors to this difference. Aromatics are relatively resistant to degradation, as cleavage of such a ring structure requires an energy-consuming process for bacteria, especially under anoxic conditions (Ismail and Gescher, 2012). However, high nutrient concentrations promote the growth and energy generation of bacteria. Therefore, the higher level of nutrients such as nitrate, ammonia, and dissolved silicate (Sharp, 1991; Zhang et al., 2007) in the bottom seawater, compared with surface seawaters, might be beneficial for bacteria to degrade these recalcitrant/semi-labile compounds (Zaidi and Imam, 1999; Carlson et al., 2002; Beolchini et al., 2010). Therefore, the C-degradation potential of the microbial community is closely associated with the composition of available organic compounds, which is in turn dependent on the depth and the freshwater discharged from the YZR.

Microarray-based technology is reasonably quantitative and has a high hybridization specificity for generating results that can be species/strain specific (Tiquia et al., 2004; He et al., 2010). A total of 312 gene families are targeted in the GeoChip 4, such as C cycling (41), N cycling (17), organic remediation (184), P utilization (3), and sulfur metabolism (6) (Tu et al., 2014). Meanwhile, in the GeoChip 4, ∼22% of probes are sequence-specific and the other ∼78% of probes are group-specific, which covers 141,995 coding sequences (Tu et al., 2014). Furthermore, the GeoChip 4 targets 4,332 bacterial strains, 188 archaeal strains, 420 eukaryotic strains, and 273 bacteriophage (Tu et al., 2014). It is available for ecosystem-level studies owing to the capability of simultaneously identifying and quantifying many microbial functional genes/pathways (Zhou et al., 2015). A study demonstrated the feasibility of using the GeoChip in a spatial ecological investigation of the marine microbial community (Yergeau et al., 2007). Since it has many probes from different functional genes, the GeoChip used in this study could detect, reasonably well, some functional genes that have been studied intensively, although continuous improvement with higher gene coverage is always needed (Zhou et al., 2008). For example, previous quantitative-PCR results indicated that the abundance of *amoA* and *nirS* was higher in the bottom than in the surface water (Zhang et al., 2014a). However, we did not observe a significant difference in the proportion of these gene groups with our GeoChip analysis. These results might associate with the different primer/probe coverage between these two methods (Braker et al., 1998; Francis et al., 2005; He et al., 2010; Huang et al., 2011; Zhang et al., 2014a). In addition, the possible underestimation of functional diversity by microarray-based hybridization should be noted if the probes on the arrays do not well-represent the diversity of the genes of interest in a given microbial community (Zhou et al., 2008). In contrast, a metagenomic approach could uncover more information from rare species, which are also crucial for the functioning of the ecosystem. Despite of the advantages and disadvantages of each approach, previous study found the consistent results of microbial community using between metagenomic and GeoChip approach in soil environment responded to climate change (Xue et al., 2016), which indicated the complementation of these approaches. In addition, the local environmental factors not measured in our study, such as the dissolved oxygen, the quantity and bioavailability of organic carbon, and the concentration and composition of N and P nutrients, might be influenced by the water masses, which might impact the functioning of the microbial community as well (Jiang et al., 2014; Gan et al., 2016). Therefore, a more comprehensive study would be needed to evaluate the influence of these attributes on the functional diversity of the microbial community in the ECS.

## Conclusion

In this study, we comprehensively examined the microbial functional gene diversity and structure in the complex ECS shelf area using GeoChip technology to understand the ecological role of water microbial communities and how that role is affected by environmental dynamics. The overall functional gene pattern of the ECS microbial communities was not consistent with their phylogenetic diversity pattern, with higher functional diversity and metabolic potential in the surface water compared with the bottom water. The microbial metabolic preference for different carbon substrates was revealed by the proportion of carbon-degradation genes. Our statistical analysis suggested that prominent environmental factors (temperature, salinity, and Chl *a*) associated with water mass were strong drivers in shaping the functional gene pattern of water microbial communities. These findings provide insight into the effects of the differing water masses on the ecological potential of microbes in the ECS. However, it should be noted that the presence of a functional gene or gene fragment does not necessarily imply functionality. Therefore, expression-based studies, such as mRNA-based microarray hybridization and metatranscriptome sequencing, are required to further elucidate microbial roles in this coastal sea ecosystem.

## Author Contributions

YW contributed for the experiment conduction and data analysis. YW and RZ contributed for the manuscript writing. ZH, JVN, and QZ contributed for the manuscript amendments on technology and language. RZ, JZ, and NJ contributed for the experiment design.

## Conflict of Interest Statement

The authors declare that the research was conducted in the absence of any commercial or financial relationships that could be construed as a potential conflict of interest.

## Footnotes

1 http://ieg.ou.edu/microarray/

2 http://ieg.ou.edu/4download/

## Abbreviations

ANOVA: analysis of variance
Chl *a*: chlorophyll *a*
ECS: East China Sea
KC: Kuroshio Current
PERMANOVA: permutational multivariate analysis of variance
TWC: Taiwan Warm Current
YZR: Yangtze River
