# Supplementary material for: Functional Gene Diversity and Metabolic Potential of the Microbial Community in an Estuary-Shelf Environment
Source: Front Microbiol. 2017 Jun 21;8:1153. doi: 10.3389/fmicb.2017.01153 (PMC5478683; doi:10.3389/fmicb.2017.01153)
Supplement: Supplementary file 1 [file Data_Sheet_1.pdf]

Supplementary figures and tables

Figure S1 Principal component analysis of environmental parameters in the ECS.

Figure S2 Detrended correspondence analysis of all probes detected in the microbial communities of the ECS.

Figure S3 Proportion of each subcategory within the stress category. Significant differences between the groups, and surface and bottom samples, indicated by ANOVA with Tukey's post-hoc test are indicated by letters a and b, and by * and $, separately, above the bars. The different letters indicate $P < 0.05$. * Indicates higher mean relative abundance in surface samples, while $ indicates higher mean relative abundance in bottom samples with $P < 0.05$.

Figure S4 Clustering analysis based on Bray-Curtis distance at the functional subcategory level among surface and bottom samples in DH1_6, DH2_6, and DH3_6 using the ward.D2 clustering method. Color bar denotes the proportion (relative abundance, %) of each subcategory. Sample name is site with depth of surface (S) or bottom (B). Only subcategories with proportion of > 1% are shown.

Table S1 Environmental parameters and total number of phylotypes that were detected for each gene of sampling stations in the East China Sea.

Table S2 Student t test of proportion of genes involved in N cycling between surface and bottom seawater.

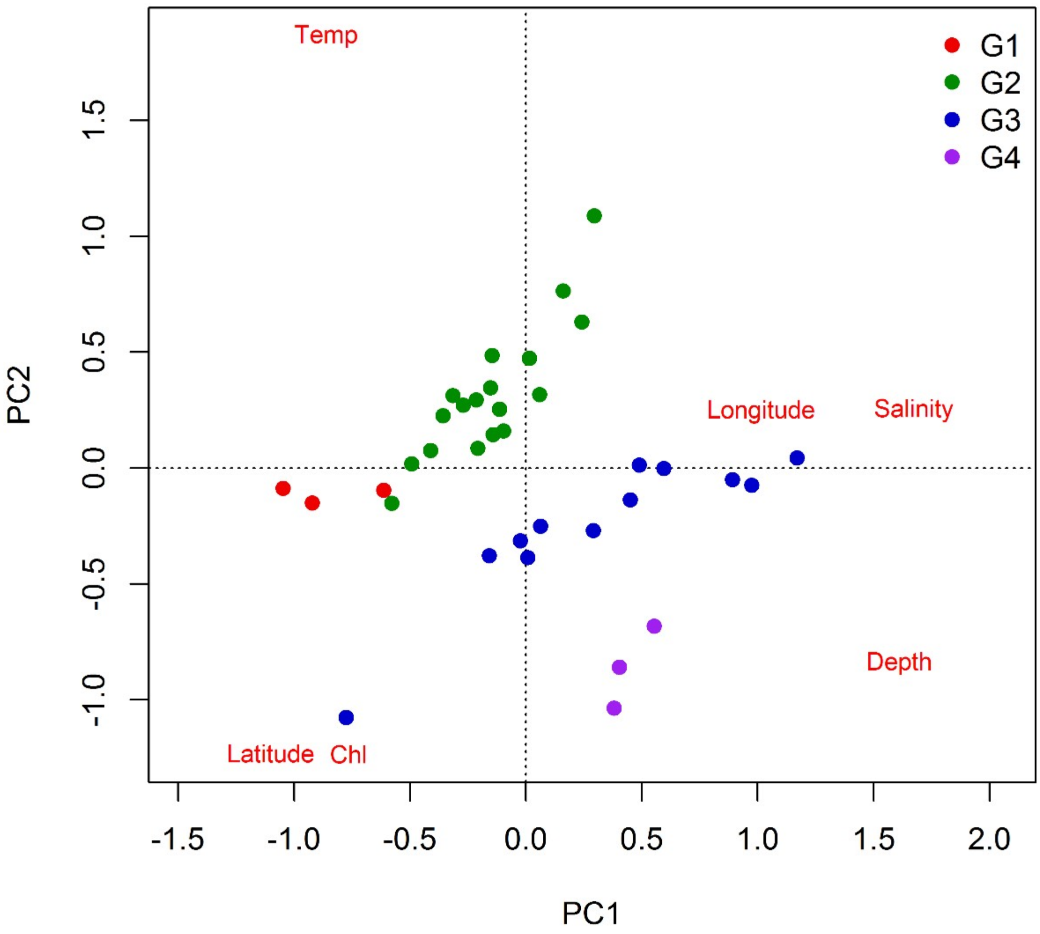

Figure S1 Principal component analysis of environmental parameters in the ECS.

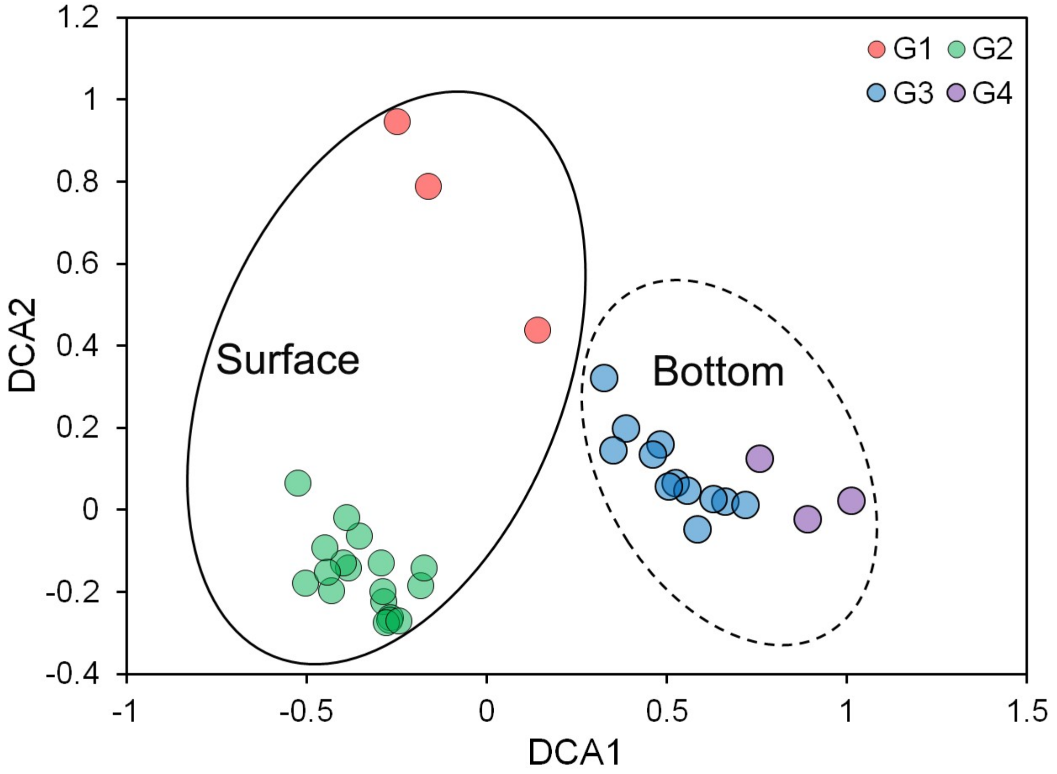

Figure S2 Detrended correspondence analysis of all probes detected in the microbial communities of the ECS.

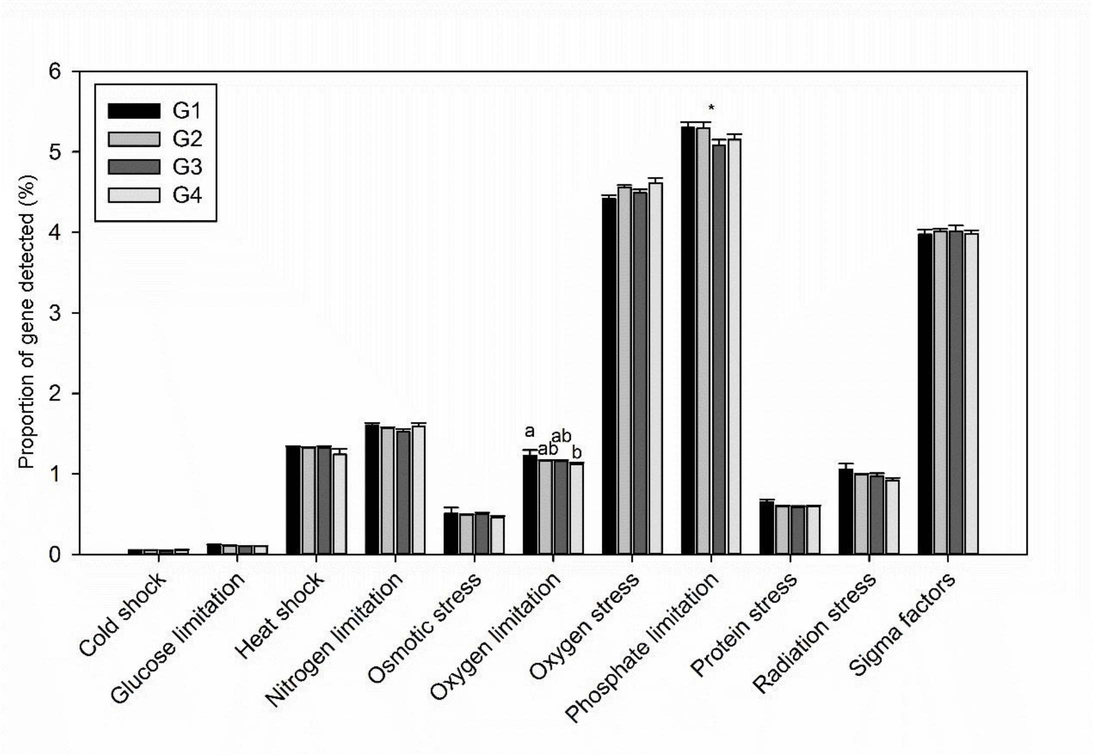

Figure 3 Proportion of gene detected in each subcategory within the stress category. Significant differences between the groups, and surface and bottom samples, indicated by ANOVA with Tukey's post-hoc test are indicated by letters a and b, and by * and $, separately, above the bars. The different letters indicate $P < 0.05$. * Indicates higher mean relative abundance in surface samples, while $ indicates higher mean relative abundance in bottom samples with $P < 0.05$.

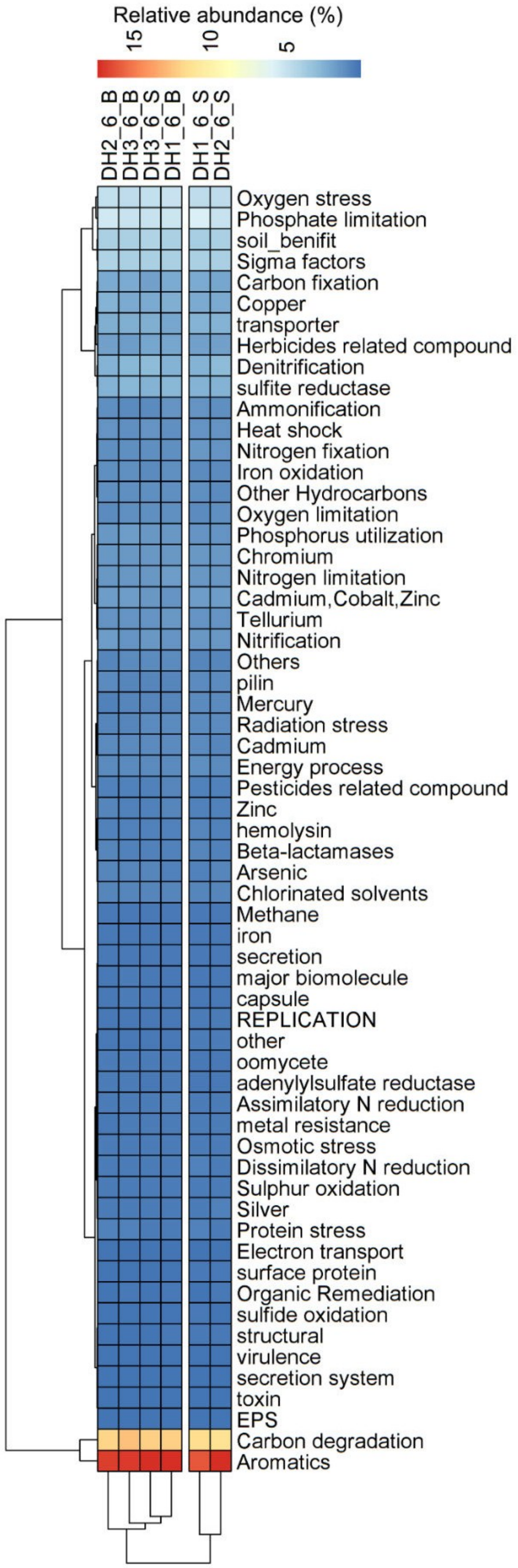

Figure S4 Clustering analysis based on Bray-Curtis distance at the functional subcategory

level among surface and bottom samples in DH1_6, DH2_6, and DH3_6 using the ward.D2 clustering method. Color bar denotes the proportion (relative abundance, %) of each subcategory. Sample name is site with depth of surface (S) or bottom (B). Only subcategories with proportion of > 1% are shown.

Table S1 Environmental parameters and total number of phylotypes that were detected for each gene of sampling stations in the East China Sea.

Group	Station	Depth (m)	Latitude (°)	Longitude (°)	Temperature (℃)	Salinity (‰)	Chl a (ppb)[a]	Gene #[b]
G1	DH2_1_S	2	31.5	122.51	21.47	18.96	1.28	10956
	DH3_1_S	2	30.96	122.57	20.6	20.33	1.87	8879
	DH4_1_S	2	30	122.5	20.46	25.69	2.81	7761
G2	DH1_1_S	2	32	122.5	20.85	28.78	1.8	5393
	DH1_3_S	2	32	123.5	21.84	28.46	1.28	8456
	DH1_6_S	2	32.01	124.97	21.69	30.47	1.11	10506
	DH2_3_S	2	31.5	123.5	22.72	30.44	0.67	9585
	DH2_6_S	2	31.5	125	21.28	30.95	0.48	14634
	DH3_3_S	2	30.99	123.51	22.19	30.59	0.21	10782
	DH3_6_S	2	31	125	21.19	29.75	1.05	14044
	DHa_2_S	2	30.52	123	20.81	28.01	1.04	11360
	DHa_4_S	2	30.5	124.01	22.12	30.11	0.61	10160
	DH4_3_S	2	30	123.5	22.01	31.47	0.45	10714
	DHa_6_S	2	30.5	124.96	21.53	29.6	0.74	11881
	DH5_1_S	2	29.47	123.1	21.5	28.01	0.39	14350
	DH4_6_S	2	30.03	125	21.15	31.58	0.39	11149
	DH5_3_S	2	29.09	123.72	22.08	32.84	0.4	10020
	DH5_6_S	2	28.42	125.04	22.51	33.65	0.5	14287
	DH6_2_S	2	28.29	122.44	22.11	31.63	0.64	10014
	DH6_5_S	2	27.55	123.61	23.08	34.08	0.13	9758
	DH6_8_S	2	26.81	124.77	25.2	34.45	0.13	12232
G3	DH3_1_B	18	30.96	122.57	19.4	28.85	11.57	6358
	DH1_1_B	25	32	122.5	18.11	31.69	0.76	5879
	DH2_1_B	27	31.5	122.51	18.18	33.4	0.81	11068
	DH2_3_B	37	31.5	123.5	18.55	31.63	0.28	4518
	DH3_3_B	52	30.99	123.51	18.28	33.61	0.44	13873
	DH1_3_B	39	32	123.5	18.03	30.93	0.51	4116
	DH5_1_B	61	29.47	123.1	18.44	34.57	0.53	14548
	DH5_3_B	76	29.09	123.72	19.57	34.45	0.31	5904
	DH5_6_B	98	28.42	125.04	18.3	34.54	0.21	13886
	DH6_2_B	66	28.29	122.44	18.85	34.54	0.24	7478
	DH6_5_B	98	27.55	123.61	18.07	34.66	0.2	8733
	DH6_8_B	115	26.81	124.77	18.37	34.68	0.11	11522
G4	DH1_6_B	45	32.01	124.97	13.53	32.3	1.44	7131
	DH2_6_B	47	31.5	125	13.23	32.54	3.75	8016
	DH3_6_B	55	31	125	14.03	32.38	0.81	8552

[a]denotes chlorophyll *a* concentration.

[b]denotes number of gene detected in each sample.

Table S2 Student t test of proportion of genes involved in N cycling between surface and bottom seawater.

Gene	Category	t value	p value
ureC	Ammonification	0.42	0.523
gdh	Ammonification	7.18	0.011
hzo	Anammox	1.52	0.227
nasA	Assimilatory N reduction	0.01	0.934
nirA	Assimilatory N reduction	0.10	0.752
NiR	Assimilatory N reduction	0.42	0.520
NirB	Assimilatory N reduction	0.00	0.953
narG	Denitrification	0.10	0.754
nirS	Denitrification	4.34	0.045
nirK	Denitrification	0.31	0.580
norB	Denitrification	1.81	0.187
nosZ	Denitrification	0.03	0.860
nrfA	Dissimilatory N reduction	0.14	0.714
napA	Dissimilatory N reduction	0.77	0.387
amoA	Nitrification	0.01	0.907
hao	Nitrification	6.89	0.013
nifH	Nitrogen fixation	4.95	0.033
